# Molecular disparities in colorectal cancers of White Americans, Alabama African Americans, and Oklahoma American Indians

**DOI:** 10.1038/s41698-023-00433-5

**Published:** 2023-08-19

**Authors:** Hiroshi Y. Yamada, Chao Xu, Kenneth L. Jones, Philip H. O’Neill, Madka Venkateshwar, Srikanth Chiliveru, Hyung-Gyoon Kim, Mark Doescher, Katherine T. Morris, Upender Manne, Chinthalapally V. Rao

**Affiliations:** 1 Department of Internal Medicine, Hematology/Oncology Section, University of Oklahoma Health Sciences Center (OUHSC), Oklahoma City, OK USA; 2 Center for Cancer Prevention and Drug Development, Stephenson Cancer Center, University of Oklahoma Health Sciences Center (OUHSC), Oklahoma City, OK USA; 3 Department of Biostatistics and Epidemiology, Hudson College of Public Health, University of Oklahoma Health Sciences Center (OUHSC), Oklahoma City, OK USA; 4 Department of Cell Biology, University of Oklahoma Health Sciences Center (OUHSC), Oklahoma City, OK USA; 5 Harold Hamm Diabetes Center, University of Oklahoma Health Sciences Center (OUHSC), Oklahoma City, OK USA; 6 Department of Pathology, Heersink School of Medicine, University of Alabama at Birmingham, Birmingham, AL USA; 7 Community Outreach and Engagement, Stephenson Cancer Center, University of Oklahoma Health Sciences Center (OUHSC), Oklahoma City, OK USA; 8 Department of Surgery, University of Oklahoma Health Sciences Center (OUHSC), Oklahoma City, OK USA; 9 VA Medical Center, Oklahoma City, OK USA

**Keywords:** Colon cancer, Cancer epidemiology, Gene expression profiling

## Abstract

In the US, the majority of cancer samples analyzed are from white people, leading to biases in racial and ethnic treatment outcomes. Colorectal cancer (CRC) incidence and mortality rates are high in Alabama African Americans (AAs) and Oklahoma American Indians (AIs). We hypothesized that differences between racial groups may partially explain these disparities. Thus, we compared transcriptomic profiles of CRCs of Alabama AAs, Oklahoma AIs, and white people from both states. Compared to CRCs of white people, CRCs of AAs showed (a) higher expression of cytokines and vesicle trafficking toward modulated antitumor-immune activity, and (b) lower expression of the ID1/BMP/SMAD axis, IL22RA1, APOBEC3, and Mucins; and AIs had (c) higher expression of PTGS2/COX2 (an NSAID target/pro-oncogenic inflammation) and splicing regulators, and (d) lower tumor suppressor activities (e.g., TOB2, PCGF2, BAP1). Therefore, targeting strategies designed for white CRC patients may be less effective for AAs/AIs. These findings illustrate needs to develop optimized interventions to overcome racial CRC disparities.

## Introduction

For cancer therapy, a “personalized medicine” approach, in which treatment is tailored to an individual’s cancer-specific mutational profile, has become increasingly common. This approach, combined with targeted medicine, can result in dramatic improvements in efficacy and survival^1^. For cancer prevention, the personalized medicine approach is applied to select high-risk groups based on genetic predisposition or lifestyle, such as carriers of the Lynch syndrome gene(s) (colorectal cancer (CRC)) or smokers (lung adenocarcinoma)^2^. As it is necessary to examine a high-risk population to identify genetic predispositions, medical practitioners need to accept a broad approach to target-specific signaling, mutations, neoantigens, and/or conditions. For personalized medicine, knowledge of the molecular events involved in cancer is essential.

Much of the current knowledge on cancer therapies is based on available cancer specimens, which, in the US and Europe, are overwhelmingly from white patients. While research data and registries are the basis of precision oncology, current biorepositories do not represent cancers in racial and ethnic minorities. For example, in a TCGA pan-cancer-based analysis in 2018^3^, the racial categorization for 170 cases of rectal adenocarcinoma was White/Black/Other/Not Applicable=82/6/1/81, and for 45 cases of cholangiocarcinoma, the categorization was 38/3/3/1. Thus, racial disparities exist in the available cancer sample size, which impedes the progression of knowledge of cancer development and therapies for non-white groups. In particular, in the United States (US), African Americans (AAs) and Asians are poorly represented in observational, translational, and clinical cancer studies, and American Indians (AIs) /Native Americans had poor or no representation. In all study designs, white people are overrepresented^4^. Although AAs are 13.4%, and AIs are 1.1% of the population in the 2020 US census^5^, cancer sample availabilities are disproportionally limited, and molecular analyses focusing on cancers in AAs or AIs are sparse.

In the US, AA and AI populations are not evenly distributed. AA populations are larger in the Southern states, including Alabama, and larger AI/Alaska Native populations reside in Alaska, Northwest states, and Southwest states, including Oklahoma^5^. As few Alaska Natives reside in Oklahoma, the term “AI” alone is used in this report. Cancer disparities in these racial/community groups have gained attention^6–8^. For instance, in a study that validated county-level mortality rates for 29 cancers, areas with high numbers of AAs in Alabama and AIs in Oklahoma suffer high incidences of and mortality from cancers of the lung, breast, kidney, and colorectum^6^. This disparity is partly explained by differences in risk factors, socioeconomic factors, and access to high-quality treatment^6^. Nevertheless, for these groups, the effects of biological differences appear to contribute to cancer disparities (e.g., breast cancer^9^).

CRC is the third most common cancer diagnosed in the US (excluding skin cancers), with predicted new cases of 106,180 (colon) and 44,850 (rectal); in 2022, it was the second most common cause of cancer deaths (predicted 52,580 deaths)^10^. Thus, CRCs remain a major target for prevention and therapy. In this study, we hypothesized the existence of molecular disparities among CRCs of white patients and high CRC incidence/mortality populations, i.e., Alabama AAs and Oklahoma AIs, and aimed to identify racial disparities for CRCs at the molecular level. We selected differentially expressed genes (DEGs) and affected pathways, as well as circulating cytokines for AAs, as potential race/community group-specific disease modifiers and most actionable molecular disparities subjects. The knowledge of molecular disparities opens possibilities for developing optimized cancer prevention and therapy approaches for these racial/ethnic groups (Alabama AAs, Oklahoma AIs).

## Results

### Transcriptomic alterations in CRCs of white patients and of Alabama AAs

Between white CRCs and Alabama AA CRCs, 561 genes (2-fold cutoff, raw *p* < 0.05) were identified as DEGs. The highest number of DEGs occurred in KEGG metabolic pathways (68 genes), followed by pathways of neurodegeneration (15 genes), MAPK signaling (14 genes), axon guidance (14 genes), Alzheimer’s disease (14 genes), cancer (12 genes), amyotrophic lateral sclerosis (12 genes), Huntington disease (12 genes), and PI3K-Akt signaling (10 genes).

**Fig. 1:**
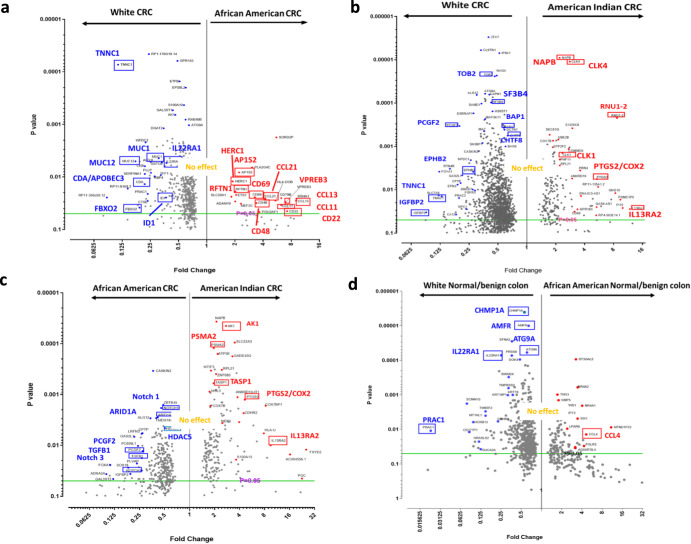
DEGs between samples from white people, AAs and AIs. **a** White CRCs vs AA CRCs. Between white CRCs and AA CRCs, 561 genes (raw *P* < 0.05) were identified as DEGs. Red dots indicate DEGs with higher expression in AA CRCs. Blue dots indicate higher expression in white CRCs. Notable DEGs are marked with a red box, including TNNC1 (troponin C1, Slow Skeletal and Cardiac Type, a muscle contraction regulator), CD69 (a T-cell activation marker), and CCL13 (C-C Motif Chemokine Ligand 13 involved in leukocyte recruitment), implicating differences in growth signaling/immune system usage. Also suggested was activation, in white CRCs, of oncogenic signaling (ID1, IL22RA1), and, in AA CRCs, a disturbance in vesicle trafficking (AP1S2, HERC1, RFTN1), which can lead to immunosuppression. **b** White CRCs vs AI CRCs. Between white CRCs and AI CRCs, 1740 genes (raw *P* < 0.05) were identified as DEGs. Notable DEGs included RNU1-2 (RNA, U1 Small Nuclear 2), IL13RA2 (Interleukin 13 Receptor Subunit Alpha 2), TNNC1 (Troponin C1, Slow Skeletal and Cardiac Type), IGFBP2 (Insulin-Like Growth Factor Binding Protein 2), and PTGS2/COX2 (a pro-oncogenic inflammation mediator and NSAID target). PTGS2/COX2 over-expression in AIs suggests use of NSAID as management strategy focused on AI CRCs. **c** AA CRCs vs AI CRCs. Between AA CRCs and AI CRCs, 611 genes (raw *P* < 0.05) were identified as DEGs. AI CRCs may utilize different pathways compared with AA CRCs; e.g., the utility of Notch signaling. In CRCs of AAs, there were a 3.76-fold decrease of Notch 3 and a 2.19-fold decrease of Notch 1 (highlighted in blue boxes). In this comparison, PTGS2/COX2 overexpression in AIs remained notable. **d** White normal/benign colonic mucosal tissue vs AA normal/benign colonic mucosal tissue. Between white naive colon vs AA naive colon, 730 genes (raw *P* < 0.05) were identified as DEGs, indicating the existence of basal non-tumor-associated DEGs in these groups. The DEGs may cause cancer proneness, thus serving as a basis for cancer predisposition and as a target for cancer prevention. Notable DEGs for AAs include CCL4 over-expression, which may lead to a pro-tumor microenvironment through recruitment of CD163+ macrophages.

Focusing on notable DEGs, Alabama AA CRCs showed (a) higher expression of immune signature and cytokines (CD69, CD48, CD22, CCL13, CCL11) and vesicle trafficking (AP1S2, HERC1, RFTN1), but (b) low expression of ID1 (BMP/SMAD axis), IL22RA1 (JAK/STAT, MAPK/MAPK3, and AKT axes), a genomic instability inducer (CDA/APOBEC3), and mucins (MUC1,12) (Fig. 1a, Supplementary Fig. S1A–C). CD69, CD48, and CD22 are the B lymphocyte or NK cell activation markers. Eosinophil attractants, CCL11 and CCL13 chemokines, are involved in allergy responses. Increases in these molecules suggest immune activation and infiltration, and a better prognosis for CRC^11^. However, given the complexities of the tumor immune system, whether an increase in these markers signifies anti-tumor or pro-tumor activity needs to be determined in the context of AA CRCs. AP1S2, a clathrin-associated adaptor protein complex subunit, is involved in the innate immune system and class I MHC-mediated antigen processing and presentation through vesicle trafficking. Vesicle trafficking is the target for human immunodeficiency virus-1 (HIV-1) NEF viral protein. NEF downregulates MHC-I by modulating the host membrane trafficking machinery, which includes AP1, HERC1, and RFTN1^12^. High expression of AP1S2 may disturb the stoichiometry of the vesicle trafficking machinery and hence may serve toward evasion of AA CRCs from host immunity. The HERC family members are involved in cancer progression; in particular, HERC1 is involved in cell migration by regulating RAF/MKK3/p38 pathways. Highly expressed RFTN1 promotes gastric cancer by modulating AKT/p38 signaling pathways^13^. The BMP/SMAD, JAK/STAT, MAPK, and AKT axes are growth signaling and there are existing drugs targeting these axes clinically available^14^. Yet, with functionally antagonizing gene expressions co-occurring (i.e., high HERC1, RFTN1 activating the growth signaling vs. low IL22RA1 leading to diminishing activation) in AA CRCs, whether the drugs will indicate efficacy predicted from white CRCs remains to be seen. High expression of CDA/APOBEC3, a mutator complex that causes pro-tumor genomic instability in breast and lung cancers^15^, is another target for ongoing cancer drug development. Alabama AA CRCs (a) may employ an immune evasion mechanism, while signs of immune activations are present, and (b) carry differences in oncogenic pathway usage that may result in poor response to treatments standardized by and for CRC of whites (see Discussion).

Pathway analysis indicated two pathways substantially affected (B-H *p*-values < 0.05); they are (i) biosynthesis of colanic acid building blocks and (ii) ephrin A signaling (Table 1A). High expression of the pathways for biosynthesis of colanic acid building blocks is associated with certain types of bacterial abundances (e.g., *Ezakiella, Clostridium sensu stricto, Porphyromonas, Barnesiella*) and co-occurring CRC symptoms^16^, suggesting possible differences in microbiota between CRC patients of white and of Alabama AAs. The ephrin A signaling pathway, which promotes malignant transformation of colorectal epithelial cells by upregulating various oncogenic signaling via lncRNAs^17^, is a proposed drug target. Yet, for AAs, ephrin A signaling genes are under-expressed (e.g., EFNA3, 0.43-fold; EFNA1, 0.57-fold; EFNA4, 0.63-fold), which may lead to poor responses of drugs targeting the ephrin A pathway.

**Table 1:** Affected pathways between white patients, AA and AI CRC samples.

Ingenuity Canonical Pathways	-log(B-H *p*-value)	(B-H) *p*-value
**(A) White vs African American, CRC**		
Colanic Acid Building Blocks Biosynthesis	2.23	0.005888437
Ephrin A Signaling	1.58	0.02630268
**(B) White vs American Indian, CRC**		
Spliceosomal Cycle	2.4	0.003981072
Autophagy	2.4	0.003981072
Germ Cell-Sertoli Cell Junction Signaling	1.67	0.021379621
Huntington’s Disease Signaling	1.67	0.021379621
14-3-3-mediated Signaling	1.67	0.021379621
Synaptic Long Term Potentiation	1.67	0.021379621
Sirtuin Signaling Pathway	1.67	0.021379621
Molecular Mechanisms of Cancer	1.66	0.021877616
Colanic Acid Building Blocks Biosynthesis	1.66	0.021877616
CLEAR Signaling Pathway	1.65	0.022387211
GDP-mannose Biosynthesis	1.63	0.023442288
PPARα/RXRα Activation	1.48	0.033113112
Granzyme B Signaling	1.45	0.035481339
Protein Kinase A Signaling	1.44	0.036307805
Ephrin B Signaling	1.44	0.036307805
Pyridoxal 5’-phosphate Salvage Pathway	1.41	0.038904514
Ephrin Receptor Signaling	1.37	0.042657952
AMPK Signaling	1.37	0.042657952
Endocannabinoid Developing Neuron Pathway	1.37	0.042657952
Sertoli Cell-Sertoli Cell Junction Signaling	1.37	0.042657952
Axonal Guidance Signaling	1.35	0.044668359
Role of OCT4 in Mammalian Embryonic Stem Cell Pluripotency	1.34	0.045708819
RAR Activation	1.34	0.045708819
Signaling by Rho Family GTPases	1.34	0.045708819
**(C) African American vs American Indian, CRC**		
B Cell Receptor Signaling	2.24	0.005754399
RAR Activation	1.53	0.029512092
IL-15 Signaling	1.53	0.029512092
Systemic Lupus Erythematosus In B Cell Signaling Pathway	1.52	0.030199517
Notch Signaling	1.52	0.030199517
EIF2 Signaling	1.52	0.030199517
Molecular Mechanisms of Cancer	1.52	0.030199517
Coronavirus Pathogenesis Pathway	1.52	0.030199517
Xenobiotic Metabolism PXR Signaling Pathway	1.47	0.033884416
mTOR Signaling	1.41	0.038904514
Huntington’s Disease Signaling	1.41	0.038904514
**(D) White normal/benign vs African American normal/benign colon**		
Granulocyte Adhesion and Diapedesis	1.71	0.019498446
Role of MAPK Signaling in Inhibiting the Pathogenesis of Influenza	1.63	0.023442288
Agranulocyte Adhesion and Diapedesis	1.63	0.023442288
Role of Hypercytokinemia/hyperchemokinemia in the Pathogenesis of Influenza	1.59	0.025703958
MIF Regulation of Innate Immunity	1.42	0.03801894
Coronavirus Pathogenesis Pathway	1.42	0.03801894

(A) Two pathways were identified as affected (B-H-*p* < 0.05), white CRCs as control and AA CRCs as treatment group. (B) Twenty-two pathways were identified as affected. (C) Eleven pathways were identified as affected. (D) Six pathways were identified as affected.

### Transcriptomic alterations in CRCs of whites and of Oklahoma AIs

Between white people and Oklahoma AIs, 1740 DEGs (2-fold, *P* < 0.05) were identified. The highest number of DEGs occurred in 159 genes that are involved in metabolic pathways, followed by pathways of neurodegeneration (47 genes), pathways in cancer (47 genes), Alzheimer disease (40 genes), salmonella infection (36 genes), amyotrophic lateral sclerosis (35 genes), human papillomavirus infection (34 genes), endocytosis (34 genes), shigellosis (32 genes), regulation of the actin cytoskeleton (31 genes), and MAPK signaling (30 genes).

AI CRCs showed, as notable DEGs, (a) higher expression of PTGS2/COX2 and splicing regulators (RNU1-2, CLK1, CLK4), but (b) lower expression of an inflammation inhibitor (TOB2), tumor suppressors (PCGF2 [polycomb group ring finger protein, which acts as repressor of genes involved in embryogenesis, the cell cycle, and tumorigenesis]; BAP1 [BRCA1-interacting RING finger protein]); a factor involved in genome stability (CHTF8 [a component of Ctf18 Replication Factor C complex]); and a motility suppressor (EPHB2) (Fig. 1b, Supplementary Fig. S1D). The profile suggests, for AI CRCs, prominent roles of COX2 and arachidonic acid-mediated pro-oncogenic inflammation^18^, splicing mis-regulation-and under-expression of CTF18-mediated genomic instability, and decreased tumor suppressor activities.

Relating to KEGG pathway analyses, in comparison with white CRCs, Oklahoma AI CRCs showed 22 pathways that were significantly different (B-H *p*-value < 0.05) (Table 1B). These pathways included the spliceosome cycle, autophagy, 14-3-3 mediated signaling, sirtuin signaling, and biosynthesis of colanic acid building blocks, suggesting new drug targets for AI CRCs.

### Transcriptomic alterations in CRCs of AAs and AIs

When the transcriptomes were compared between CRCs from AA to those from AI, highest numbers of DEGs occurred in metabolic pathways (38 genes), followed by pathways in cancer (18 genes), pathways of neurodegeneration (15 genes), human papillomavirus infection (15 genes), endocytosis (14 genes), Huntington disease (13 genes), coronavirus disease (12 genes), Parkinson disease (12 genes), Alzheimer disease (12 genes), and Prion disease (11 genes).

Although both Alabama AAs and Oklahoma AIs are identified with high CRC risk, their transcriptome profiles showed characteristic differences, indicating racial molecular disparities between AAs and AIs. AI CRCs (a) utilized pro-oncogenic inflammatory signaling (PTGS2/COX2) more than AA CRCs and (b) depended less on Notch (Notch1, Notch3) and chromatin remodeling (ARID1A, HDAC5) (Fig. 1c). IL13RA2 was overexpressed in AI CRCs. This molecule is an immune-related DEG for papillary renal cell carcinoma with prognostic value^19^. Pathway analysis revealed notable DEGs; the list includes B cell receptor signaling, RAR activation, IL15 signaling, Notch signaling, and EIF2 signaling (Table 1C).

### Transcriptomic alterations in normal/benign colonic epithelial tissues of white people and of AA

DEGs in normal/benign colon tissues may be relevant to cancer predisposition and may serve as targets for cancer prevention. For normal/benign colon tissues of white patients, PRAC1 (prostate cancer susceptibility candidate 1) and endosomal sorting and autophagy genes (CHMP1A, AMFR, ATG9A), and IL22RA1 were highly expressed; they are proposed as targets for therapy^20–22^. However, the expression levels of these genes were lower in normal/benign colon tissues of AAs (Fig. 1d), suggesting that targeting of these molecules will be useful for CRC prevention and treatment of white patients but will have limited utility for those of AAs. Notably, for AAs, CCL4 was highly expressed (Supplementary Fig. S1E). CCL4 in CRCs induces the infiltration of tumor-associated pro-tumor macrophages (CD163+ cells)^23^, suggesting that a CCL4 inhibitor may have a chemopreventive effect for AA CRCs through modulation of the microenvironment.

Pathway analyses indicated that affected pathways are mostly associated with immune functions and infection responses, such as granulocyte adhesion and diapedesis, the role of MAPK signaling in inhibiting the pathogenesis of influenza, agranulocyte adhesion and diapedesis, the role of hypercytokinemia/hyperchemokinemia in the pathogenesis of influenza, MIF regulation of innate immunity, and the coronavirus pathogenesis pathway (Table 1D).

### Circulating inflammatory cytokines in white and AA patients

**Fig. 2:**
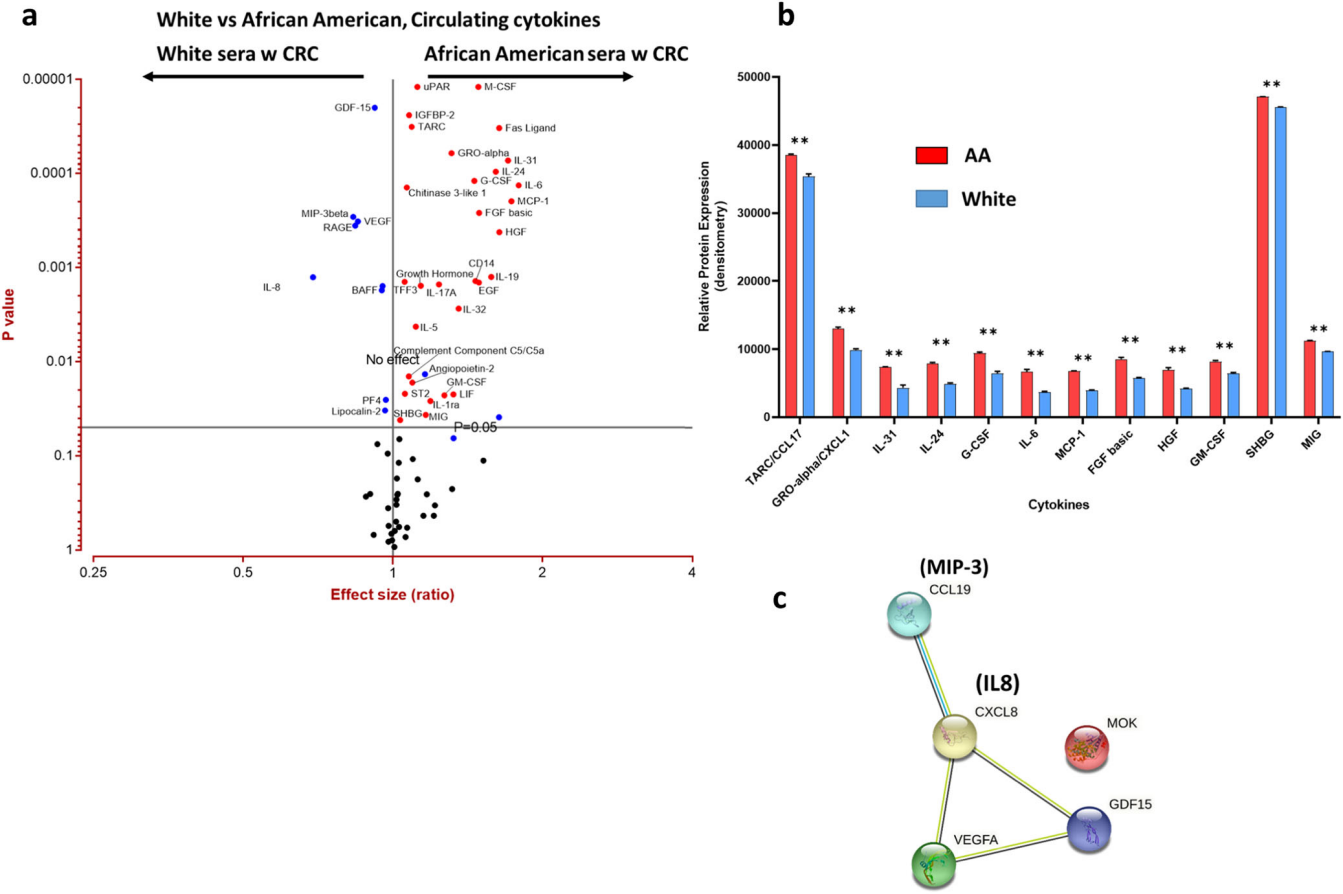
Assays for serum circulating cytokine indicates higher expressions of IL8-associated cytokines in white CRC patients compared with AA CRC patients. **a** Volcano plot for cytokine ELISA. Notably, MIP3, VEGF, IL8, GDF15 and RAGE are found higher in white patients’ sera. **b** Twelve cytokines identified as differentially expressed (*p* < 0.05). **c** IL8-GDF15-VEGFA axis (String interaction map). CCL19, CXCL8, VEGFA and GDF15 are all functionally linked. CCL19 is identical to MIP3, and CXCL8 is identical to IL8. Error bars based on standard deviation (S.D.).

Based on DEGs involved in immune functions being higher in AA CRCs, we hypothesized that there would be differences in circulating cytokines in sera of AA CRC patients. ELISA panel analysis for 105 cytokines indicated, for AA CRC patients, higher expressions (1.5-fold, *p* < 0.001) of 12 cytokines: CCL17, CXCL1, IL31, IL24, G-CSF, IL6, MCP1, FGF basic, HGF, GM-CSF, SHBG, and MIG (Fig. 2a, b).

Notably, in samples obtained from white patients, increased expression of GDF15, MIP3 (CCL19), VEGF, IL8 (CXCL8), and RAGE were identified (Fig. 2a). The IL-8 (CXCL8)/GDF-15/VEGFA are components of the same axis, as in string interaction map (Fig. 2c). Lower expression of the IL-8 (CXCL8)/GDF-15/VEGFA axis components in AAs suggested under-utilization of this pro-tumorigenic axis. IL-8 is a proinflammatory chemokine associated with neutrophil chemotaxis and is proposed to be a cancer therapy target. Yet, the proposal was based on data from white patients, and targeting of this axis might prove less effective for AA CRC patients. The downregulation of MIP-3beta (CCL19) is functionally correlated as it plays a suppressive role in colorectal tumorigenesis^24^. CCL19 is significantly low-expressed in CRC tissues and positively related to highly tumor micro-vessel density. CCL19 can inhibit CRC angiogenesis through promoting miR-206, thus inhibiting Met/ERK/Elk-1/HIF-1α/VEGF-A pathway^25^. The downregulation of RAGE (receptor for advanced glycation end-product) is functionally related to CRC progression. RAGE activation leads to progression and prolongation of CRC and correlates well with the survival of CRC cells^26^. Overexpression of RAGE has been associated with chronic inflammation and increased CRC risk^27^.

## Discussion

Currently, available AA and especially AI CRC samples for molecular analysis are limited. The US NIH-NCI Genomic Data Commons (GDC) database (https://gdc.cancer.gov) is among the most comprehensive data curation sites to date, including samples from GENIE, FM, TCGA, CPTAC, HCMI and EXCEPTIONAL_RESPONDERS. As of May 3, 2023, even in the GDC database that carries data from a total of 8136 (4271 white CRCs and 498 AA) CRCs, only 11 AI or Alaska Native groups CRC samples are available. Moreover, among the 11 AI samples, only two samples are with transcript profiling data. For AA, less than 82 samples are available with transcriptome data. Hence, this study represents a valuable study albeit with limited number of samples. However, future studies with large sample sets are warranted to enable researchers to further analyze covariates and to build mathematical prediction models.

The present study showed differences in DEGs and pathway usage among CRCs of white patients, AAs, and AIs. Ethnic/racial groups may share biological patterning that occurs as a result of social constructs, as well as dietary habitats, geographic locations, and other cultural practice and lifestyles^6^. It will not be simple to associate these co-occurring variates with causation of diseases. Yet, the study identifies several molecular differences, some of which are druggable and/or actionable, that may contribute to racial disparities in CRCs among Alabama AAs and Oklahoma AIs. In many cases, a gene being a DEG is a part of rationale for selecting target genes for drug development. This molecular analysis study points out that DEG profiles are different among CRCs from white patients, Alabama AAs and Oklahoma AIs. Thus, it will serve to raise awareness to pay attention to racial/ethnicity or community-based DEGs. It is to be noted that some of the significantly altered DEG (Ex; COX-2) in AAs and AIs already had FDA approved drugs, thus immediately actionable. These findings lend support for the development of race/ethnicity-optimized CRC therapy and prevention strategies.

This study also provides cautionary results, which suggest that proposed drug development targets based on the data obtained from CRCs of white patients may not be as applicable to CRCs of AAs or AIs. Targeting of growth signaling (SMAD, STAT, MAPK, AKT axes) and genomic instability (APOBEC3 mutator) are strategies that are being pursued, and these drugs under development may be employed as next generation biologics. Yet, the molecular target rationalization is based on studies conducted on mostly white patients with CRC. Our transcriptomic analysis implicates reduced efficacy of these “new standard” strategies for CRCs of AAs.

Regarding the therapeutic efficacy aspect, lower 5-year survival rates of CRCs in AAs have been well-documented. For example, Alese et al. ^28^ investigated CRC in younger (age 18-50) population and reported that “despite equally receiving standard of care as per national guidelines, AA had significantly lower 5-year survival rates (58.8%) compared to Hispanics (64.8%) and Non-Hispanic White patients (66.9%; HR 1.42; 1.38–1.46; *p* < 0.001).” As mentioned in the introduction, regional Alabama AA data indicated the same tendency. A meta-analysis in 2011 concluded that Black patients with resected stage II and stage III CRC who were treated with the same therapy as white patients experienced worse overall and recurrence-free survival compared with white patients. To explain the disparity in survival, a possibility that Black patients may have a genetically unique response or toxicity to treatment compared with white patients was discussed^29^.

In a recent US national CRC cohort study with a focus on race/ethnicity, lower overall survival rate for American Black CRC patients compared with American white CRC patients was confirmed, and multitudes of domains were identified to explain the lower survival in AAs; clinical stage at presentation, time of surgery, access to minimally invasive surgery, post-surgical outcomes, access to chemotherapy, and cumulative incidence of death^30^.

In another study focusing on metastatic CRC^31^, Black and white CRC patients treated with chemotherapy (i.e., cytotoxic drugs; 5-fluorouracil, capecitabine, oxaliplatin, and irinotecan) and biologics (bevacizumab, cetuximab, panitumumab, ramucirumab, and aflibercept) indicated no significant difference in the survival benefits^31^. The authors of the study^31^ concluded that survival benefits of biologics are not significantly different between white patients and AA patients. However, as overall survival rates are rather low with stage 4 CRCs patients (14-15%) indicating that overall therapeutic efficacy was limited across populations tested, we suggest to interpret the data with caution and to consider possibility of different applicability of the conclusion to earlier stage CRCs. The biologics included in the analysis were not directly targeting the DEGs in CRCs in this study. As such, differences between racial groups playing a role in efficacy of therapy remain as valid scenario.

Nevertheless, the present study results provide promising targets. For Oklahoma AIs, high PTGS2/COX2, as a notable DEG, suggests that COX2 inhibition through NSAIDs might serve as a feasible and implementable modality for CRC prevention. Mis-regulation in the splicing factor complex can lead to genomic instability^32^, and generation of tumor neoantigens through splice site misuse^33^. Thus, studies to identify neoantigens abundant in AI CRCs may prove fruitful for developing an AI-focused CRC vaccine. Additionally, as biological and molecular differences are more actionable at the medical practice level than socioeconomic factors, biological disparities applicable at the community level should be investigated/explored for translational purposes. The outcomes of molecular analysis may lead to new sets of biomarkers (a) that are race/ethnicity-specific and (b) can be integrated to current biomarker-driven personalized medicine practice.

Overall, our findings provide a proof-of-principle toward the approach of “personalized medicine at a community level,” and may enable researchers to design and optimize race/ethnicity- or community-specific CRC prevention and/or therapy programs.

### Limitations and recommendations

Since no AI normal/benign colon samples or sera were collected, we were unable to compare them with samples from white people or AAs. The number of Oklahoma AI CRC samples was small; as sample procurement for Oklahoma AIs remains challenging for this and other projects that rely on tissue specimens. A promising solution would be to develop one or more regional biorepositories for AI tissue samples. This would involve collaboration between cancer centers and Tribal Nations, and, once developed, would provide a means of creating databases with larger numbers of tumor samples from AIs. Governance procedures for standardized data/sample sharing procedures for these biorepositories will need to be developed and followed.

## Methods

### Samples

We procured DNA and RNA samples, collected during surgery, from 20 white patients (CRC and normal/benign; *N* = 40) and 20 AA patients (*N* = 40) (total 80 RNA and 80 DNA samples), as well as sera from 12 white CRC patients and 8 AA patients (from Dr. U. Manne, UAB). Samples were de-identified, other than racial information, which was not shared with data analysts until analyses were completed. Measurements were taken from distinct samples. Except for samples with quality issues (e.g., DNA/RNA degradation), all samples were included and analyzed. Due to the de-identification, some of co-variates analysis was not conducted. We also procured CRC tissue samples from prospectively consented patients (*n* = 7, white and *n* = 6, AI) (from Dr. K.T. Morris, OUHSC, Surgery Department). We followed sample collection, procurement, and analysis protocols approved by UAB and OUHSC, as appropriate. Authors have written informed consent from participants under the OUHSC-approved IRB protocol #7565. The PI of the protocol is Dr. Morris. Race/ethnicity categorization (White, Black or African American, American Indian or Alaska Native, Asian, and Native Hawaiian or Other Pacific Islander) was provided by researchers in accordance with guidelines provided by the U.S. Office of Management and Budget (OMB), and these data are based on participants’ self-identification.

### Bulk RNAseq

Tissue RNA and DNA were extracted with Trizol (ThermoFischer/Invitrogen) and stored at −80 ^o^C until use. DNA and RNA samples were submitted to the OUHSC Institutional Research Core Facility. Stranded RNA-seq libraries were constructed using NEBNext poly(A) mRNA isolation kit with the SWIFT RNA Library Kit and the established protocols. Exome libraries were built using Agilent’s SureSelect XT HS2 Library and Target Enrichment Kit following Agilent’s established protocols. Results of the ongoing DNA sequencing will be described elsewhere. The library construction was accomplished with 500 ng of RNA or 4 ng–200 ng of DNA. During library construction, each of the libraries was indexed in order to multiplex for sequencing. Samples were normalized and pooled onto a 150 paired end run on Illumina’s NextSeq 2000. Illumina Seq paired fastq files were aligned in Strand NGS software version 2.1 (www.strand-ngs.com) using human assembly. Reads were normalized using DESeq. The normalized read counts were log-transformed and base-lined to the data set, resulting in normalized signal values. Differential gene expression of the normalized signal values between the control and experimental group was determined with a 2-fold cutoff using a moderated *t*-test, *p* < 0.05. The differentially expressed gene list was subsequently used for clustering and pathway analysis.

We used Student’s *t*-test to analyze the data. Statistical significance was evaluated by algorithms integral to the aforementioned software. FDR-adjusted *p*-values of < 0.05 were considered significant. Figures were generated with Graphpad Prism 9.40 software using integrated algorithms.

### Cytokine assay with ELISA

Proteome Profiler Human XL Cytokine Array (Cat# ARY022B, R&D Systems, Minneapolis, MN, USA) was used to analyze the expression of 105 human secretory cytokines in patient plasma samples. Nitrocellulose membranes pre-coated with capture antibodies were incubated overnight at 2−8 ^o^C with 200 µl of plasma sample diluted in a buffered protein base according to the manufacturer’s protocol. Subsequently, membranes were incubated with a biotinylated detection antibody cocktail and antibody conjugation with streptavidin-HRP was followed by the recommended washes. The membranes were developed using Chemi Reagent Mix and signal detection was accomplished with ChemiDoc Imaging software (Bio-Rad, Hercules, CA, USA). Signal intensity (densitometry) was quantified using Quick Spots/ HLImage + + software (Ideal Eyes Systems, UT, USA).

### Reporting summary

Further information on research design is available in the Nature Research Reporting Summary linked to this article.

### Supplementary information

Supplementary Information

Reporting Summary

## Data Availability

The bulk RNA sequencing data generated in this study have been submitted to Gene Expression Omnibus (GEO) (NIH, US) and will be publicly available at GSE237684 upon publication of this manuscript. Alternatively, all relevant data are available from the authors on reasonable request.

## References

[1] Cercek A (2022). PD-1 Blockade in Mismatch Repair-Deficient, Locally Advanced Rectal Cancer. N. Engl. J. Med..

[2] Meyskens FL (2015). Cancer Prevention: Obstacles, Challenges and the Road Ahead. J. Natl. Cancer Inst..

[3] Liu J (2018). An Integrated TCGA Pan-Cancer Clinical Data Resource to Drive High-Quality Survival Outcome Analytics. Cell.

[4] Behring M (2019). Inclusiveness and ethical considerations for observational, translational, and clinical cancer health disparity research. Cancer.

[5] US Census 2020 https://www.census.gov/programs-surveys/decennial-census/decade/2020/2020-census-results.html (2020).

[6] Mokdad AH (2017). Trends and Patterns of Disparities in Cancer Mortality Among US Counties, 1980 2014. JAMA.

[7] Campbell J (2016). Five-Year Cancer Survival Rates in Oklahoma from 1997 to 2008. J. Okla. State Med. Assoc..

[8] Albano JD (2007). Cancer mortality in the United States by education level and race. J. Natl. Cancer Inst..

[9] Martini R (2022). African Ancestry-Associated Gene Expression Profiles in Triple-Negative Breast Cancer Underlie Altered Tumor Biology and Clinical Outcome in Women of African Descent. Cancer Discov..

[10] American Cancer Society: Cancer Facts and Figures 2022. American Cancer Society, 2022. https://www.cancer.org/research/cancer-facts-statistics/all-cancer-facts-figures/cancer-facts-figures-2022.html (2022).

[11] Korbecki J (2020). CC Chemokines in a Tumor: A Review of Pro-Cancer and Anti-Cancer Properties of the Ligands of Receptors CCR1, CCR2, CCR3, and CCR4. Int J. Mol. Sci..

[12] Dirk BS (2016). HIV-1 Nef sequesters MHC-I intracellularly by targeting early stages of endocytosis and recycling. Sci. Rep..

[13] Deng C (2022). RFTN1 facilitates gastric cancer progression by modulating AKT/p38 signaling pathways. Pathol. Res Pr..

[14] Narayanankutty A (2019). PI3K/ Akt/ mTOR Pathway as a Therapeutic Target for Colorectal Cancer: A Review of Preclinical and Clinical Evidence. Curr. Drug Targets.

[15] Venkatesan S (2021). Induction of APOBEC3 Exacerbates DNA Replication Stress and Chromosomal Instability in Early Breast and Lung Cancer Evolution. Cancer Discov..

[16] González-Mercado VJ (2021). Co-Occurrence of Symptoms and Gut Microbiota Composition Before Neoadjuvant Chemotherapy and Radiation Therapy for Rectal Cancer: A Proof of Concept. Biol. Res. Nurs..

[17] Papadakos SP, Petrogiannopoulos L, Pergaris A, Theocharis S (2022). The EPH/Ephrin System in Colorectal Cancer. Int J. Mol. Sci..

[18] Ballerini P (2022). Inflammation and Cancer: From the Development of Personalized Indicators to Novel Therapeutic Strategies. Front. Pharm..

[19] Wang L, Gu W, Ni H (2021). Construction of a prognostic value model in papillary renal cell carcinoma by immune-related genes. Med. (Baltim.).

[20] Barnicle A, Seoighe C, Greally JM, Golden A, Egan LJ (2017). Inflammation-associated DNA methylation patterns in epithelium of ulcerative colitis. Epigenetics.

[21] Devenport SN, Shah YM (2019). Functions and Implications of Autophagy in Colon Cancer. Cells.

[22] McCuaig S (2020). The Interleukin 22 Pathway Interacts with Mutant KRAS to Promote Poor Prognosis in Colon Cancer. Clin. Cancer Res..

[23] De la Fuente López M (2018). The relationship between chemokines CCL2, CCL3, and CCL4 with the tumor microenvironment and tumor-associated macrophage markers in colorectal cancer. Tumour Biol..

[24] Lu L (2014). Antitumor Efficacy of CC Motif Chemokine Ligand 19 in Colorectal Cancer. Dig. Dis. Sci..

[25] Xu Z (2018). CCL19 suppresses angiogenesis through promoting miR-206 and inhibiting Met/ERK/Elk-1/HIF-1α/VEGF-A pathway in colorectal cancer. Cell Death Dis..

[26] Azizian-Farsani F (2020). Receptor for Advanced Glycation End Products Acts as a Fuel to Colorectal Cancer Development. Front Oncol..

[27] Aglago EK (2021). Soluble Receptor for Advanced Glycation End-products (sRAGE) and Colorectal Cancer Risk: A Case–Control Study Nested within a European Prospective Cohort. Cancer Epidemiol. Biomark. Prev..

[28] Alese OB (2019). Analysis of racial disparities in the treatment and outcomes of colorectal cancer in young adults. Cancer Epidemiol..

[29] Yothers G (2011). Outcomes among black patients with stage II and III colon cancer receiving chemotherapy: an analysis of ACCENT adjuvant trials. J. Natl. Cancer Inst..

[30] Greenberg AL (2023). Exploring the complexity and spectrum of racial/ethnic disparities in colon cancer management. Int J. Equity Health.

[31] Goel S, Negassa A, Acuna-Villaorduna A (2021). Comparative Effectiveness of Biologic Agents Among Black and White Medicare Patients in the US With Metastatic Colorectal Cancer. JAMA Netw. Open.

[32] Tam AS, Stirling PC (2019). Splicing, genome stability and disease: splice like your genome depends on it!. Curr. Genet..

[33] Sahin I, George A, Seyhan AA (2021). Therapeutic Targeting of Alternative RNA Splicing in Gastrointestinal Malignancies and Other Cancers. Int J. Mol. Sci..

