# Supplementary material for: Molecular disparities in colorectal cancers of White Americans, Alabama African Americans, and Oklahoma American Indians
Source: NPJ Precis Oncol. 2023 Aug 19;7:79. doi: 10.1038/s41698-023-00433-5 (PMC10439889; doi:10.1038/s41698-023-00433-5)
Supplement: Supplementary file 1 — Supplementary Information [file 41698_2023_433_MOESM1_ESM.pdf]

Supplementary Information file

Supplementary Figure 1. Normalized gene expression data from the RNAseq for notable DEGs.

(A) CCL13 in white CRCs and AA CRCs. CCL13 is overexpressed in AAs. **(B) CDA/APOBEC3 in white CRCs and AA CRCs.** CDA/APOBEC3, a mutator gene, is overexpressed in white CRCs but not in AA CRCs. **(C) MUC1 in white CRCs and AA CRCs.** MUC1, a target for CRC vaccine development, is over-expressed in white CRCs but not in AA CRCs, pointing to lower vaccine efficacy in AAs. **(D) PTGS2/COX2 in white CRCs and AI CRCs.** The NSAID target PTGS2/COX is overexpressed in AI CRCs, presenting an actionable target for AI CRCs prevention and/or management with NSAIDs. **(E) CCL4 in white normal/benign colons and AA normal/benign colons.** CCL4 is over-expressed in AA normal/benign colons. CCL4 induces infiltration of pro-tumor CD163+ macrophages, leading to a pro-tumorigenic microenvironment, and serving, for AAs, as a potential target for prevention and inhibition of CRCs. *** $P<0.05$.

Supplementary Figure 1

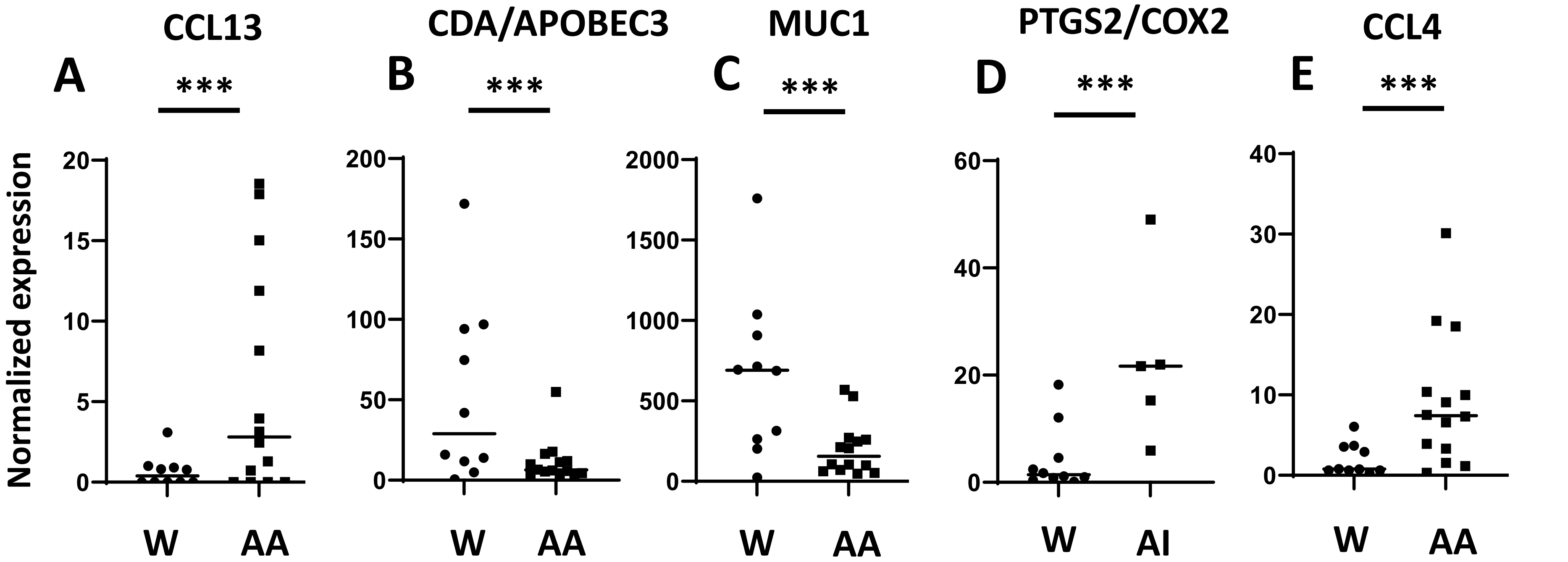

White CRCs vs AA CRCs

White CRCs vs AI CRCs

White normal/benign vs AA normal/benign
