# Supplementary material for: Molecular disparities in colorectal cancers of White Americans, Alabama African Americans, and Oklahoma American Indians
Source: NPJ Precis Oncol. 2023 Aug 19;7:79. doi: 10.1038/s41698-023-00433-5 (PMC10439889; doi:10.1038/s41698-023-00433-5)
Supplement: Supplementary file 2 — Reporting Summary [file 41698_2023_433_MOESM2_ESM.pdf]

nature portfolio

Corresponding author(s): *Double-anonymous peer review submissions: write DAPR and your manuscript number here instead of author names.*

Last updated by author(s): *YYYY-MM-DD*

Reporting Summary

Nature Portfolio wishes to improve the reproducibility of the work that we publish. This form provides structure for consistency and transparency in reporting. For further information on Nature Portfolio policies, see our Editorial Policies and the Editorial Policy Checklist.

Statistics

For all statistical analyses, confirm that the following items are present in the figure legend, table legend, main text, or Methods section.

n/a	Confirmed	
☐	☒	The exact sample size (*n*) for each experimental group/condition, given as a discrete number and unit of measurement
☐	☒	A statement on whether measurements were taken from distinct samples or whether the same sample was measured repeatedly
☐	☒	The statistical test(s) used AND whether they are one- or two-sided<br>*Only common tests should be described solely by name; describe more complex techniques in the Methods section.*
☐	☒	A description of all covariates tested
☐	☒	A description of any assumptions or corrections, such as tests of normality and adjustment for multiple comparisons
☐	☒	A full description of the statistical parameters including central tendency (e.g. means) or other basic estimates (e.g. regression coefficient) AND variation (e.g. standard deviation) or associated estimates of uncertainty (e.g. confidence intervals)
☐	☒	For null hypothesis testing, the test statistic (e.g. *F*, *t*, *r*) with confidence intervals, effect sizes, degrees of freedom and *P* value noted<br>*Give P values as exact values whenever suitable.*
☒	☐	For Bayesian analysis, information on the choice of priors and Markov chain Monte Carlo settings
☒	☐	For hierarchical and complex designs, identification of the appropriate level for tests and full reporting of outcomes
☒	☐	Estimates of effect sizes (e.g. Cohen's *d*, Pearson's *r*), indicating how they were calculated

Our web collection on statistics for biologists contains articles on many of the points above.

Software and code

Policy information about availability of computer code

Data collection	NA
Data analysis	Reads were normalized using DESeq. The normalized read counts were log-transformed and base-lined to the data set, resulting in normalized signal values. Differential gene expression of the normalized signal values between the control and experimental group was determined with a 2-fold cutoff using a moderated t test, p < 0.05.

For manuscripts utilizing custom algorithms or software that are central to the research but not yet described in published literature, software must be made available to editors and reviewers. We strongly encourage code deposition in a community repository (e.g. GitHub). See the Nature Portfolio guidelines for submitting code & software for further information.

Data

Policy information about availability of data

All manuscripts must include a data availability statement. This statement should provide the following information, where applicable:

- Accession codes, unique identifiers, or web links for publicly available datasets
- A description of any restrictions on data availability
- For clinical datasets or third party data, please ensure that the statement adheres to our policy

Data availability statement. The bulk RNA sequencing data generated in this study have been submitted to Gene Expression Omnibus (GEO) (NIH, US) and will be publicly available at GSE237684 upon publication of this manuscript. Alternatively, all relevant data are available from the authors on reasonable request.

Research involving human participants, their data, or biological material

Policy information about studies with human participants or human data. See also policy information about sex, gender (identity/presentation), and sexual orientation and race, ethnicity and racism.

Reporting on sex and gender	Sex and gender information was not provided/available.
Reporting on race, ethnicity, or other socially relevant groupings	We procured DNA and RNA samples, collected during surgery, from 20 White patients (CRC and normal/benign; N=40) and 20 AA patients (N=40) (total 80 RNA and 80 DNA samples), as well as sera from 12 White CRC patients and 8 AA patients (from Dr. U. Manne, UAB). Samples were de-identified, other than racial information, which was not shared with data analysts until analyses were completed. Measurements were taken from distinct samples. Due to the de-identification, some of co-variates analysis was not conducted. We also procured CRC tissue samples from prospectively consented patients (n=7, White and n=6, AI) (from Dr. K.T. Morris, OUHSC, Surgery Department). We followed sample collection, procurement, and analysis protocols approved by UAB and OUHSC, as appropriate. Authors have written consent from participants under the approved IRB protocol #7565. The PI of the protocol is Dr. Morris.
Population characteristics	Population characteristics information was not provided/available.
Recruitment	We procured DNA and RNA samples, collected during surgery, from 20 White patients (CRC and normal/benign; N=40) and 20 AA patients (N=40) (total 80 RNA and 80 DNA samples), as well as sera from 12 White CRC patients and 8 AA patients (from Dr. U. Manne, UAB). Samples were de-identified, other than racial information, which was not shared with data analysts until analyses were completed. Measurements were taken from distinct samples. Due to the de-identification, some of co-variates analysis was not conducted. We also procured CRC tissue samples from prospectively consented patients (n=7, White and n=6, AI) (from Dr. K.T. Morris, OUHSC, Surgery Department). We followed sample collection, procurement, and analysis protocols approved by UAB and OUHSC, as appropriate. Authors have written consent from participants under the approved IRB protocol #7565. The PI of the protocol is Dr. Morris. Race/ethnicity categorization (White, Black or African American, American Indian or Alaska Native, Asian, and Native Hawaiian or Other Pacific Islander) was provided by researchers in accordance with guidelines provided by the U.S. Office of Management and Budget (OMB), and these data are based on participants' self-identification.
Ethics oversight	We followed sample collection, procurement, and analysis protocols approved by UAB and OUHSC, as appropriate. Authors have written consent from participants under the approved IRB protocol #7565. The PI of the protocol is Dr. Morris.

Note that full information on the approval of the study protocol must also be provided in the manuscript.

Field-specific reporting

Please select the one below that is the best fit for your research. If you are not sure, read the appropriate sections before making your selection.

☒ Life sciences ☐ Behavioural & social sciences ☐ Ecological, evolutionary & environmental sciences

For a reference copy of the document with all sections, see nature.com/documents/nr-reporting-summary-flat.pdf

Life sciences study design

All studies must disclose on these points even when the disclosure is negative.

Sample size	Since no AI normal/benign colon samples or sera were collected, we were unable to compare them with Whites or AAs. The number of Oklahoma AI CRC samples was small; as sample procurement for Oklahoma AIs remains challenging for this and other projects that rely on tissue specimens.<br>For single test, a two-sided, two-sample t-test with group sample sizes of 6 and 6 achieves 80% power to detect a fold change of 2.0 when the fold change under the null hypothesis is 1.000, assuming the coefficient of variation on the original scale is 0.4 and the significance level (alpha) is 0.05. If we consider multiple companions, we will need far more samples.
Data exclusions	Except for samples with quality issues (e.g., DNA/RNA degradation), all samples were included and analyzed.
Replication	For single test, a two-sided, two-sample t-test with group sample sizes of 6 and 6 achieves 80% power to detect a fold change of 2.0 when the fold change under the null hypothesis is 1.000, assuming the coefficient of variation on the original scale is 0.4 and the significance level (alpha) is 0.05. If we consider multiple companions, we will need far more samples.
Randomization	Samples were de-identified, other than racial information, which was not shared with data analysts until analyses were completed.
Blinding	Samples were de-identified, other than racial information, which was not shared with data analysts until analyses were completed.

Reporting for specific materials, systems and methods

We require information from authors about some types of materials, experimental systems and methods used in many studies. Here, indicate whether each material, system or method listed is relevant to your study. If you are not sure if a list item applies to your research, read the appropriate section before selecting a response.

Materials & experimental systems

n/a	Involved in the study
☒	☐ Antibodies
☒	☐ Eukaryotic cell lines
☒	☐ Palaeontology and archaeology
☒	☐ Animals and other organisms
☒	☐ Clinical data
☒	☐ Dual use research of concern
☒	☐ Plants

Methods

n/a	Involved in the study
☒	☐ ChIP-seq
☒	☐ Flow cytometry
☒	☐ MRI-based neuroimaging
